# Gulf War illness with or without post-traumatic stress disorder: differential symptoms and immune responses

**DOI:** 10.1186/s40779-023-00508-1

**Published:** 2024-01-12

**Authors:** Faith Nguyen, Ashok K. Shetty

**Affiliations:** Department of Cell Biology and Genetics, School of Medicine, Institute for Regenerative Medicine, Texas A&M University Health Science Center, 1114 TAMU, 206 Olsen Boulevard, College Station, TX 77843 USA

**Keywords:** Gulf War Illness, Immune response, Interleukin-15, Natural killer cells, Post-traumatic stress disorder, T lymphocytes

Over one-third of 700,000 military personnel who served in the first Gulf War (GW) suffer from an assortment of symptoms, including cognitive and memory problems, musculoskeletal pain, gastrointestinal discomfort, fatigue, and respiratory issues [1, 2]. The precise etiology of Gulf War illness (GWI) is unclear. However, epidemiological and preclinical studies imply that exposures to the prophylactic drug pyridostigmine bromide, insecticides, pesticides, smoke from oil well fires, and interaction between these exposures and war-related stress underlie this illness [2]. As per the Kansas case definition, GWI is a chronic multi-symptom illness displaying one moderately severe and/or multiple symptoms of any severity in at least three of six symptom domains (fatigue, pain, neurological/cognitive/mood, skin, gastrointestinal, respiratory) [1, 2]. While the exact pathophysiological changes underlying GWI have not been identified, alterations in immune regulation and dysregulation of the redox balance have been observed in GWI, resulting in chronic systemic inflammation and neuroinflammation [2].

Post-traumatic stress disorder (PTSD) is a trauma-associated disorder that typically transpires in some individuals who underwent or viewed a petrifying, awful, or threatening event [3]. The symptoms comprise 1) re-experiencing in the form of recollections, nightmares, and terrifying thoughts; 2) avoidance typified by emotional detachment, avoidance of events, places, or objects; 3) hyperarousal distinguished by tensed feeling, being easily startled, and sleeping difficulties; and 4) cognitive and/or mood impairments indicated by difficulty remembering the traumatic event, anhedonia, undesirable views about oneself and the world [4]. PTSD is slightly more frequent in veterans (approximately 7%) than civilians (approximately 6%). However, the frequency of PTSD is much higher (approximately 35%) in GW veterans [5]. The presence of PTSD makes the diagnosis of GWI difficult because of many analogous symptoms and pathophysiological alterations.

A study by Sultana et al. [5] attempted to dissociate differential symptoms and immune cell responses in GWI with or without PTSD. The study comprised three cohorts of male GW veterans from Miami and Boston with or without GWI. Based on PTSD-related symptoms, the GWI group was further subdivided into with high or low probability of PTSD symptoms subgroups (GWI_H_ and GWI_L_). The study employed multiple measures to assess symptom scales. Analysis of the blood samples comprised a complete blood cell count, cytokine analysis, flow cytometry, and natural killer (NK) cell cytotoxicity. Data were analyzed separately for the three cohorts; only one completed the exercise challenge. Compared with veterans without GWI, veterans in both GWI_H_ and GWI_L_ subgroups displayed worse symptoms, with the GWI_H_ subgroup exhibiting the most severe symptoms. Interestingly, compared with veterans without GWI (healthy control group), the concentration of interleukin-15 (IL-15) was lower in the GWI_L_ subgroup but not in the GWI_H_ subgroup. The populations of CD3^+^CD56^+^ NK cells reduced in both GWI_H_ and GWI_L_ subgroups, but the differences did not reach statistical significance. However, the population of CD2^+^CD26^+^ cells increased by approximately 15% at peak exercise in veterans of the GWI_L_ subgroup. While both GWI subgroups displayed a reduced percentage of basophils, the GWI_L_ subgroup exhibited reduced NK cell activity compared with GWI_H_ subgroup [5]. The GWI_L_ subgroup from the Miami cohort also displayed reduced IL-15 following exercise challenge compared with GWI_H_ subgroup.

The study by Sultana et al. [5] uncovered several significant findings aiding the differentiation of GWI with or without PTSD. First, while basophils reduced in both GWI_L_ and GWI_H_ subgroups at peak exercise, the reduction was greater in GWI_L_ subgroup. As basophils regulate T cells to mediate immune response, lower levels support an altered immune profile in GWI. However, it remains to be investigated whether lower basophil numbers at peak exercise could be a biomarker of GWI compared with healthy sedentary veterans. The second important finding is that GWI_L_ veterans displayed lower IL-15 levels (approximately 50–60%) than GWI_H_ and healthy control group. Such reduced IL-15 level could potentially serve as a biomarker of GWI, as reduced IL-15 level was apparent at both rest and post-exercise in GWI_L_ veterans (i.e., GWI subgroup without PTSD). Additionally, this subgroup displayed reduced NK cell activity and NK cell surface antigens (CD3^−^CD56 or CD3^−^CD16). These results are consistent with the role of IL-15 in the development, differentiation, and survival of NK cells [5, 6]. However, deficiency in NK cells in GWI_L_ group was only significant at peak exercise. CD26 regulates T, B, myeloid, and NK cells, and abnormal CD26 levels have been observed in autoimmune diseases. Because of inter-relationships between CD2^+^CD26^+^ cells, IL-15, and NK cells, elevated levels of CD2^+^CD26^+^ cells typically mean higher levels of IL-15 and NK cell activity. The lack of such observation in the study by Sultana et al. [5] suggests a dysregulated immune response in GWI. Overall, the findings from the GWI_L_ subgroup in this study re-iterated that PTSD is not the underlying factor for the development and clinical presentation of GWI.

Why IL-15 level did not differ between the GWI_H_ and healthy control group is intriguing. One reason for this could be the association of PTSD with elevated IL-15 level, implying that the occurrence of PTSD comorbidity in veterans with GWI prevents the decreased IL-15 level, compared with veterans with GWI alone. Such observations highlight the need for further investigations on IL-15 level and NK cells in the immunological dysfunction associated with GWI and PTSD. One of the limitations of this study, also identified by the authors, is the lack of a PTSD-alone subgroup. The inclusion of a PTSD-alone subgroup in future studies would allow the analysis and differentiation between different biomarkers to further dissociate GWI without PTSD from GWI associated with PTSD. Furthermore, the exercise challenge was applied to only one cohort in the study. Such studies in larger cohorts of veterans with GWI would be critical to understanding differential responses of GWI_L_ and GWI_H_ subgroups and confirming a dysregulated immune response in GWI.

## Data Availability

All data needed to evaluate the conclusions of this commentary are present in the article.

## Abbreviations

GW: Gulf War
GWI: Gulf War illness
GWI_H_: Gulf War illness with high probability of PTSD symptoms
GWI_L_: Gulf War illness with low probability of PTSD symptoms
IL-15: Interleukin-15
PTSD: Post-traumatic stress disorder
NK: Natural killer
